# Recent Advances in Nanotechnology for the Treatment of Melanoma

**DOI:** 10.3390/molecules26040785

**Published:** 2021-02-03

**Authors:** Roberta Cassano, Massimo Cuconato, Gabriella Calviello, Simona Serini, Sonia Trombino

**Affiliations:** 1 Department of Pharmacy, Health and Nutritional Sciences, University of Calabria, Arcavacata di Rende, 87036 Cosenza, Italy; roberta.cassano@unical.it (R.C.); massimo.cuconato@gmail.com (M.C.); 2 Department of Translational Medicine and Surgery, Section of General Pathology, School of Medicine and Surgery, Università Cattolica del Sacro Cuore, Largo F. Vito, 00168 Rome, Italy; gabriella.calviello@unicatt.it (G.C.); simona.serini@unicatt.it (S.S.); 3 Fondazione Policlinico Universitario A. Gemelli IRCCS, Largo F. Vito, 00168 Rome, Italy

**Keywords:** melanoma, nanotechnology, nanomaterials, polymers, immunotherapy, target therapy

## Abstract

Melanoma is one of the most aggressive forms of skin cancer, with few possibilities for therapeutic approaches, due to its multi-drug resistance and, consequently, low survival rate for patients. Conventional therapies for treatment melanoma include radiotherapy, chemotherapy, targeted therapy, and immunotherapy, which have various side effects. For this reason, in recent years, pharmaceutical and biomedical research has focused on new sito-specific alternative therapeutic strategies. In this regard, nanotechnology offers numerous benefits which could improve the life expectancy of melanoma patients with very low adverse effects. This review aims to examine the latest advances in nanotechnology as an innovative strategy for treating melanoma. In particular, the use of different types of nanoparticles, such as vesicles, polymers, metal-based, carbon nanotubes, dendrimers, solid lipid, microneedles, and their combination with immunotherapies and vaccines will be discussed.

## 1. Introduction

The incidence of melanoma has been increasing over the past 50 years reaching more than 160,000 new cases worldwide each year [1,2]. The most common types of melanomas arise in skin, with only about five percent of them developing in extracutaneous sites, such as uvea, leptomeninges, or mucous membranes lining respiratory, gastrointestinal, or urogenital tracts [3,4]. Even though melanoma represents only the minimal portion (about 1%) of cutaneous malignant neoplasms, it is responsible for most of the skin cancer-associated deaths, having a high mortality rate and a high metastatic potential [5,6].

This cancer develops from melanocytes, the cells specialized in the production of the pigment melanin, which is responsible for the color of skin, hair, and eyes. Most melanomas are black or brown in color, although areas with different colors may sometimes be present [7]. Recognized risk factors for melanoma are familiarity, ultraviolet (UV) radiation exposure, and skin phenotype [8]. Among them, UV exposure represents the most potentially modifiable risk factor, and for this reason has received the greatest attention [9]. The association between exposure to UV (both UVA and UVB) and melanoma risk explains also why light-skinned people, who have low levels of melanin in skin, tolerate the exposure to UV radiations less than dark-skinned people and show a higher risk of developing melanoma. However, melanoma can affect any skin type, and that may be related to the existence of predisposing genetic backgrounds in some patients [10]. About 10% of patients have been reported to have a family history of melanoma [8]. In recent years, a series of genes harboring melanoma-predisposing mutations have been identified, but it is believed that other contributory genes remain to be discovered [10].

The outcome of melanoma is greatly influenced by the stage of the disease at presentation that is defined by several factors, such as the thickness of the lesion, the depth of invasion of the neighboring tissues, and the degree of lymph node invasion, together with the presence of metastases in other districts [11]. An excellent prognosis is usually reported for those patients that are treated during the earliest development stage, when skin melanoma affects only the superficial skin layers. However, early detection of melanoma, especially when it is not located at skin level, is hampered by the lack of appropriate tumor markers and the absence of clinically-significant symptoms until the disease reaches an advanced level [12,13]. Instead, as the neoplasm invades the surrounding tissues or other body districts, the treatment becomes very difficult and the prognosis is usually very poor, and the recently-reported 5-year relative survival rate of patients with metastatic melanoma still amounts to 25% [14].

Depending on the stage of the disease, as well as the location of the tumor and the general health conditions of the patients, different therapeutic options are currently available. There are those established and used since a long time ago, such as the surgical tumor removal, and the treatments with conventional chemotherapic drugs or radiations. However, the success of these treatments has been always very limited, ensuring only a short protection from the disease along the time [4]. For chemotherapy, in particular, this was often due to the development of resistance towards drugs such as Placlitaxel, platinum or dacarbazin, used instead with a higher degree of success for the first-line therapy of other kinds of tumors [15].

In more recent times, this has led to spend a great deal of effort in finding possible alternative therapeutic approaches, and plenty of research has explored the possibility to develop new therapeutic strategies to more precisely target cancer cells and save normal cells, as well as to reduce the undesired chemotherapy side-effects [16,17,18,19,20,21,22,23]. The application of nanotechnologies represents a recently developed strategy for improving the effect of both the classic or innovative pharmacological antineoplastic treatments [24]. In particular, considerable progress has been achieved by the use of nanoparticles (NPs), which represent the most recent development in the field of drug delivery. It has been observed that NPs can reach tumor cells with a high specificity and precision thanks to their components and extremely small sizes [25,26]. They are constructed with the aim to be able to easily exit the vessel wall to reach the target tumor more directly and specifically as compared to the active principles carried by them. This allows a more efficient accumulation inside tumor cells [27]. In addition, in some cases, it has been reported that they are able to offer excellent protection against enzymatic degradation or immune attack [28,29,30]. Finally, NPs may contain factors able to down-regulate oncogenes or restore tumor suppressor genes [31,32,33].

There exists a large body of recent literature focused on NPs as effective drug carriers for an innovative strategy appliable to the treatment of melanoma. The aim of this review is to describe and critically analyze the main results obtained in this field.

## 2. Passive and Active Drug Targeting Mediated by Nanoparticles

A more efficient drug delivery to the tumor, especially through NPs, can be achieved both passively and actively [34,35]. Thanks to these mechanisms, that will be clarified in the next paragraphs, it is possible to reduce toxic side-effects, increase efficacy, and enhance delivery of poorly soluble or sensitive therapeutic molecules.

### 2.1. Passive Targeting

**Figure 1:**
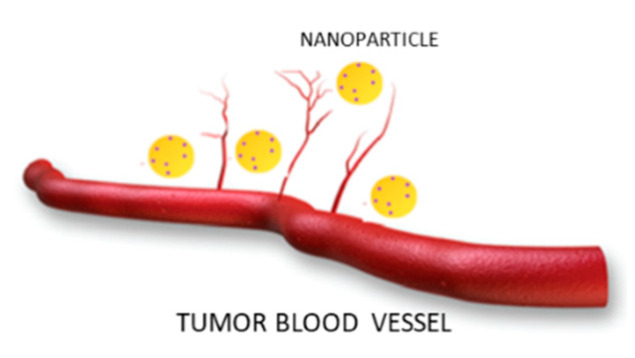
Behavior of nanoparticles in the blood vessels of the tumor area.

The peculiar characteristics of tumor neo-vascularization are at the basis of the nanotechnology-based drug delivery systems. Tumor blood vessels differ greatly from those of healthy tissues because they are discontinuous and own nano holes. Therefore, the NPs can exit the circulation and enter the tumor interstitial space (Figure 1). In addition, the drug accumulation in pathological area is also facilitated by the absence of lymphatic filtration.

Overall, this mechanism is known as the enhanced permeability and retention (EPR) effect and allows a passive targeting by NPs [36]. The high permeability and the improvement of the retention processes permit an efficient accumulation of NPs in solid tumors and consequently more direct release of therapeutic agents into malignant cells [37,38,39].

### 2.2. Active Targeting

**Figure 2:**
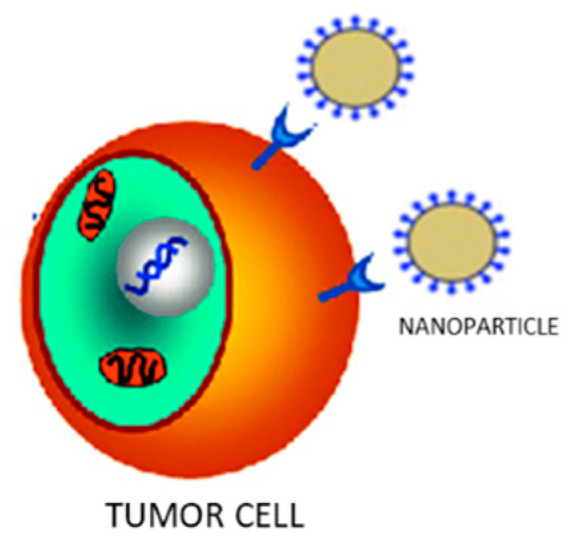
Receptor interaction between drug-containing nanoparticles and tumor cell.

Active targeting (also called ligand-based targeting) represents another interesting strategy that exploits the interactions between drug-containing NPs and tumors, to confer more specificity to the delivery system and so reducing unwanted non-specific interactions and drug localization in peripheral tissues. In particular, ligands present on the NPs surface are able to recognize receptors located on the plasma membranes of specific target cells and bind with high affinity to the receptor site [40]. Such ligands include monoclonal antibodies, peptides, and nucleic acids (Figure 2).

## 3. Nanoparticles Useful for Melanoma Treatment

Various natural or synthetic nanoparticles are valuable tools for designing new therapies for melanoma treatment: Vesicular carriers such as liposomes and niosomes, polimeric nanoparticles, inorganic ones, and carbon nanotubes and dendrimers. Each of these nanomaterials will be described in detail.

### 3.1. Vesicular Systems

**Figure 3:**
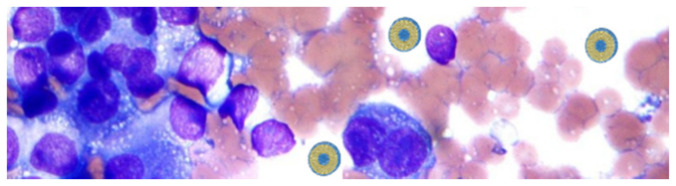
Liposomes internalization into melanoma cells.

Liposomes and niosomes are vesicular systems made up of one or more concentric bilayers useful for targeted delivery of drugs as they are able to localize the drug at the site of action, thereby lowering the drug concentration at other sites in the body. In addition, thanks to their structure, with a central aqueous compartment surrounded by a hydrophobic bilayer structure of phospholipids (liposomes) or surfactants (niosomes), they can carry both hydrophilic drugs (through encapsulation in their central compartment) and fat-soluble drugs (incorporated into the phospholipid bilayer). Furthermore, it is possible to increase their drug circulation times through various mechanisms, as size regulation or functionalization, for example with a hydrophilic agent such as polyethylene glycol (PEG). This eludes the detection by the immune system increasing circulation time and reducing toxicity of loaded drug [41,42] (Figure 3).

In 2019, Lee et al. [43] realized chitosan-coated liposomes as a formulation strategy to stabilize and enhance skin permeation of Indocyanine green (ICG), a promising candidate for the topical melanoma photodynamic therapy (PDT). The obtained liposomes protected ICG from degradation as compared to uncoated substance. Furthermore, they significantly increased cellular uptake and photocytotoxicity of ICG in B16-F10 melanoma cells in a chitosan-dependent manner. Skin permeation of ICG was also drastically improved by chitosan-coated liposomes. These results highlight the promising potential of ICG-loaded chitosan-coated liposomes for topical PDT of melanoma.

In the same year Mishra and coworkers formulated hyaluronic acid (HA)-coated liposomes and loaded with an effective combination of Dacarbazine and Eugenol two anti-melanoma agents, through a solvent injection method [44]. Coated-Dacarbazine Eugenol Liposomes showed 95.08% cytotoxicity at a dacarbazine concentration of 0.5 µg/mL with respect to dacarbazine solution that showed only 10.20% cytotoxicity at the same concentration. Furthermore, migration assay and proliferation studies also revealed significantly higher inhibition of cell migration and proliferation by Coated-Dacarbazine Eugenol Liposomes. This behavior is supposed to be due to the inhibition of the anti-apoptotic protein surviving, which is overexpressed in the melanoma cells, and makes them resistant towards apoptosis. Including Eugenol resulted in a downregulation of survivin protein, so permitting to dacarbazine to perform its function with a significantly higher cytotoxicity, increased apoptosis, and much decreased migration and proliferation of the cancer cells. Thus, surface-functionalized Dacarbazine- and Eugenol-loaded liposomes hold great promise against resistant and aggressive metastatic melanoma.

Rata et al. obtained AS1411 aptamer-functionalized liposomes for the treatment of basal cell carcinomas (BCC). AS1411 aptamer is a 26-mer DNA aptamer that specifically binds to nucleolin, a ~76 KDa protein highly expressed in the plasma membrane of different cancer cells including melanoma but is not present in normal cells [45].

Rata and coworkers, have already studied the AS1411 aptamer-functionalized polymeric nanocapsules for BCC therapy [46] and on the basis of obtained results they decided to realize aptamer-functionalized liposomes. In particular, aptamer moieties increased the stability of the liposomes and acted as a supplementary steric barrier leading to a lower cumulative amount of the released 5-Fluorouracil and reducing the surface potential of the liposomes. The in vitro cell viability, targeting capability, and apoptotic effects of liposomes on the human dermal fibroblasts and on BCC cell lines were also evaluated. The results indicated that the functionalized liposomes, as nanocapsules [47] are more efficient as nanocarriers than the non-functionalized ones.

In their study Chermahini and Najaf obtained niosomes to deliver fluorouracil to deep part of skin without any side effects and with enhanced permeability [48]. These niosomes could be potentially useful in the elimination of skin cancer cell. For this purpose, after encapsulation of fluorouracil inside the noisomes, their size and stability was measured by Malvern Mastersizer. The result showed that the size was between 200 to 400 nm and the zeta potential was about 60 indicating a good stability. The obtained results suggested that niosomes affected the permeation of fluorouracil into the skin.

Very interesting is also the work of Cristiano et al. that achieved particular ultradeformable vesicles namely, ethosomes^®^ and transfersomes^®^, for the incapsulation of sulforaphane a natural dietary isothiocyanate with in vitro antiproliferative activity against melanoma and other skin cancer diseases [49]. Unfortunately, this compound cannot be applied in free form on the skin due to its poor percutaneous permeation determined by its physico-chemical characteristics. For this reason, the authors evaluated ethosomes^®^ and transfersomes^®^ as carriers for the percutaneous delivery of sulforaphane and their physico-chemical features were assessed. They showed mean sizes <400 nm and a polydispersity index close to 0. The stability studies demonstrated that the most suitable ultradeformable vesicles to be used as topical carriers of sulforaphane were ethosomes^®^ made up of ethanol 40% (*w*/*v*) and phospholipon 90G 2% (*w*/*v*). Furthermore, permeation studies, through human stratum corneum and epidermis membranes, showed an increase of the percutaneous permeation of sulforaphane. The antiproliferative activity of sulforaphane-loaded ethosomes^®^ was tested on SK-MEL 28 cell lines and improved anticancer activity was observed in comparison with the free drug.

### 3.2. Polymeric Nanoparticles

Polymeric nanoparticles (NP) have been developed with the aim of minimizing the loss and premature degradation of the drug included within them, which usually follows the chemical and/or enzymatic deactivation. They have shown to have the potential to increase the bioavailability of the drug, reduce its harmful side effects, and increase the fraction of drug accumulated in a certain area of the organism [50]. Since most of the anticancer drugs that have been developed for melanoma are lipophilic drugs, their antitumor efficacy are limited due to their unfavorable pharmacokinetic and pharmacodynamic profiles [51]. The introduction of amphiphilic polymers (consisting of both hydrophobic and hydrophilic portions) for the formulation of anticancer drugs has successfully modified the release profile of the free drugs. Various types of polymer NPs can be synthesized depending on the properties of the polymer and their applications, namely nanospheres and nanocapsules, polymer micelles, polymers, dendrimer-based micelles, and polymer drug conjugates [40]. Very recently, Alves Batista and collaborators proposed the use of NPs, based on poly(methyl methacrylate) (PMMA) [52], with the ability to incorporate α-terpineol, a monoterpenoid known in the literature for exerting beneficial effects against leukemic cell lines [53]. The results suggest that α-terpineol increases its antitumor efficacy when incorporated into the PMMA/α-terpineol NPs and tested in melanoma-derived tumor cell lines. In addition, it was not observed any toxicity in normal cells (human macrophages and MRC-5 human fibroblasts) indicating that this formulation may be very useful to reduce the side-effects induced by many antineoplastic drugs administered in their free forms.

Scopel et al. have realized hybrid nanoparticles, composed of a PLGA core and a lipid mixture constituted by hydrogenated soy phosphatidylcholine (HSPC), cholesterol (CHOL) and 1,2-disteroyl-sn-glycero-3-phosphaethanolamine-N[succinyl(polyethyleneglycol)-2000(DSPE PEG2000) as a shell, functionalized with Vitamin D3 [54]. In particular, the aim of the work was to target vitamin D receptors (VDR) expressed in melanoma cells and so to have the possibility to convey anticancer drugs to these cells in sito-specific manner.

In vitro drug release studies showed an initial burst release in the first 24 h and after a diffusive transport. Finally, cellular uptake experiments in B16 mouse melanoma cells, demonstrated that the HNP-VDs efficiently targeted B16 melanoma cells, thus resulting in a promising vehicle to deliver therapeutics for the melanoma treatment.

Recently Cordeiro and collaborators provided to the simultaneous encapsulation of a hydrophilic drug, sodium diethyldithiocarbamate (DETC), and a hydrophobic drug, 4-nitrochalcone (4NC), in beeswax nanoparticles (BNs) with the aim to evaluate the in vitro synergic activity of these drugs against melanoma (B16F10) cells [55]. So BNs were prepared by water/oil/water double emulsion in the absence of organic solvents. This technique permitted to obtain the simultaneous encapsulation of DETC and 4NC with load efficiencies of 86.2% and 98.7%, respectively. Transmission electron microscopy imaging and dynamic light scattering analyses confirmed the formation of the particles with a semispherical shape, average diameter below 250 nm, relatively narrow distributions, and negative zeta potential. Furthermore, DETC and 4NC loaded in BNs exhibited a higher cytotoxicity toward B16F10 cells than free drugs. The simultaneous encapsulation led to a synergic effect decreasing the cell viability from 46% (DETC BNs) and 54% (4NC BNs) to 64% (DETC + 4NC BNs). The IC50 of the two drugs was lower than that of either when individually encapsulated, and respect to free DETC or 4NC. Therefore, on the basis of obtained results the simultaneous encapsulation of DETC and 4NC in BNs can be considered an important strategy against melanoma.

### 3.3. Noble Metals-Based Nanoparticles

Also biocompatible nanomaterials based on noble metals such as gold, silver, and platinum have important applications in the diagnosis and treatment of melanoma [40]. Gold NPs (AuNPs) have various biomedical advantages, such as synthetic accessibility, versatility in size, shape or the surface characteristics, biocompatibility and stability, and surface plasmon resonance [56,57]. In this regard in 2020 biocompatible gold nanoparticles (GNPs) coated with hyaluronic acid and oleic acid were prepared by Lopes and collaborators [58] and characterized in terms of size, morphology and cytotoxicity through two cell lines: The keratinocytes (healthy skin cells, HaCat) and the melanoma cells (B16F10). Results showed that these GNPs absorb within the near-infrared region (750–1400 nm), in the optical therapeutic window (from 650 to 1300 nm), in contrast to other commercial gold nanoparticles, which enables light to penetrate into deep skin layers. GNPs showed a spherical morphology with a mean size of 297 nm without cytotoxic effects towards the tested cell lines. After laser irradiation, a reduction of 20% in B16F10 cell line viability was still observed. Therefore, this seems to be a promising strategy for the treatment, in particular, of non-metastatic melanoma or other superficial tumors.

Shanei and co-authors evaluated the effect of the size of gold nanoparticles conjugated with folic acid (F-Cys-GNPs) on acoustic cavitation, a phenomenon that can be fatal for cancer cells and that occurs when a liquid is irradiated with high intensities of ultrasonic irradiation [59]. The studies conducted by Shanei et al. highlighted that the ultrasonic intensity threshold required to obtain acoustic cavitation can be considerably reduced in the presence of nanoparticles in a liquid and, in particular, their results revealed that the size increase of the F-Cys-GNPs can enhance their therapeutic effect in combination with an adequate irradiation. At the same time, it is possible to also obtain a reduction of side effects by localizing the treatment to cancerous cells thanks to acid folic acid targeting whose receptor is overexpressed in melanoma cells. The obtained results indicated that the viability of melanoma cells decreased with higher nanoparticles concentrations and sizes. Therefore, the acoustic cavitation with appropriate amount and size of GNPs can improve the therapeutic effects.

Capanema and collaborators designed and synthesized hybrid hydrogels made of silver nanoparticles (AgNPs) embedded a carboxymethylcellulose (CMC)-based hydrogel conjugated with doxorubicin (DOX), an anticancer drug [60]. This system was synthesized by a green process, using an in situ reduction of Ag+ by CMC polymer, which also acted as capping ligand, followed by the electrostatic conjugation with DOX, with the formation of colloidal nanocomplexes in aqueous media. The AgNP@CMC-DOX nanostructures, demonstrated tuned DOX intracellular kinetics in vitro, evidencing a synergistic effect with AgNPs for killing melanoma cancer cells. Moreover, they proved antimicrobial activity against Gram-positive and Gram-negative bacteria. Therefore, the obtained idrogel could be applied as a weapon against skin cancer via topical drug delivery chemotherapy.

Interesting is also the work of Valenzuela-Salas et al., about the antiproliferative and antitumor effect of AgNPs coated with polyvinylpyrrolidone (PVP) [61]. Studies of cell viability, induction of apoptosis and necrosis, and ROS generation were executed on B16-F10 cells after six hours of exposure to AgNPs or Cisplatin. Despite the similar response for both AgNPs and Cisplatin on antiproliferative potency and ROS production, significantly different cell death pathways were triggered. In particular, while AgNPs induced only apoptosis, cisplatin induced apoptosis and necrosis at the same rate. In addition, in vivo experiments exhibited the remarkable antitumor activity of a nongenotoxic AgNP formulation and representing the first advance toward the application of these AgNPs for melanoma treatment, which could considerably reduce adverse effects provoked by currently applied chemotherapeutics.

Salehi et al. evaluated the cytotoxicity of a platinum mesoporous nanostructure (Pt MN) toward a melanoma cancer cell line upon combined laser radiation and X-ray irradiation [62]. Pt MN was characterized by mesoporous structure due to the presence of ensembles of very small adhered particles of <11 nm and about 5-nm pores. The Pt MN effectively killed cancer C540 (B16/F10), in a dose dependent manner and for laser light radiation, X-ray irradiation, or for their combined exposure. Laser radiation followed by X-ray irradiation, led to a deep cell killing and a very low melanoma cell viability (~1%). Particularly, melanoma cancer cell killing by Pt MN was due to reactive oxygen species (ROS) production as a result of combined exposure to laser and X-ray, while cell killing for only laser light radiation was due to heat generation.

Mukherjee and co-workers reported the synthesis of poly ethylene glycol (PEG) assisted colloidal platinum nanoparticles (PtNPs) by borohydride reduction method at room temperature [63]. PtNPs were stable at room temperature for more than 2 years and also in a serum and phosphate buffer (pH = 7.4) solution for one week. Furthermore, these particles showed biocompatibility in different normal cell lines (in vitro) and in a chicken egg embryonic model (ex vivo). In addition, the anticancer drug doxorubicin was encapsulated in the particles. These last exhibited inhibition of cancer cell (B16F10 and A549) proliferation, observed by different in vitro assays. The PtNPs containing doxorubicin were able to induce apoptosis in cancer cells. Moreover, their intraperitoneal (IP) administration gave a considerable reduction of tumor growth in subcutaneous murine melanoma tumor model compared to control group with free drug. Therefore, on the basis of in vitro and in vivo results, platinum nanoparticle-based encapsulating doxorubicin, could be represent a therapeutic alternative for melanoma therapy.

### 3.4. Carbon Nanotubes

Carbon nanotubes (CNTs) are molecular tubes formed from one or more coiled sheets of graphene (a single layer of carbon atoms). They are classified as single-walled (SWNT) and multi-walled nanotubes (MWNT) [40]. It is possible to modify their surface by attaching other chemical groups to the tip or side wall, and it is very essential for their applications. In fact, in the last years CNTs have become increasingly important due to their biocompatibility, and potential to deliver large cargos of drugs and biomolecules [64].

**Figure 4:**
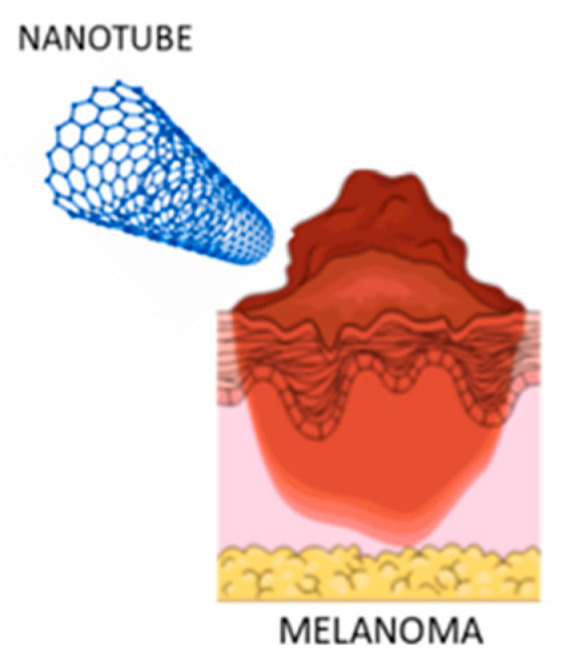
Interaction between carbon nanotubes (CNTs) and melanoma.

For all these reasons CNTs are potentially useful also for the delivery of drugs in the melanoma treatment (Figure 4).

Chaudhuri et al. [65] modified carbon nanotubes (CNTs) to conjugate a prodrug of doxorubicin (CNT-Dox) via a carbamate linker to cause a prolonged release of the active drug. In particular, the CNT-Dox induced cell death in B16-F10 melanoma cell lines in vitro. The nanovector was rapidly internalized in the lysosome of melanoma cells and was retained in the subcellular compartment for over 24 h. In an in vivo model of melanoma, treatment with the CNT-Dox abrogated tumor growth without the systemic side effects associated with free DOX. Recently, de Carvalho Lima and coworkers [66] proposed the possibility to manipulate the CNTs with peculiar physicochemical characteristics as a single platform with diagnostic and therapeutic potential for melanoma.

In a very recent study, Behzadpour et al. described the synthesis and characterization of a polypyrrole-coated multi-walled carbon nanotubes composite (PPy@MWCNTs) as a new sonosensitizer useful for the treatment of melanoma. It seems that low-intensity ultrasound irradiation accompanied by a sonosensitizer has remarkable advantages for cancer therapy such as targeted uptake, access to deeper tumors, insignificant side effects and invasiveness, compared with other therapeutic methods. Histologic analyses and tumor volume decrement after 10 days revealed 75% necrosis and 50% decrement in tumor volume [67]. 

### 3.5. Dendrimers

**Figure 5:**
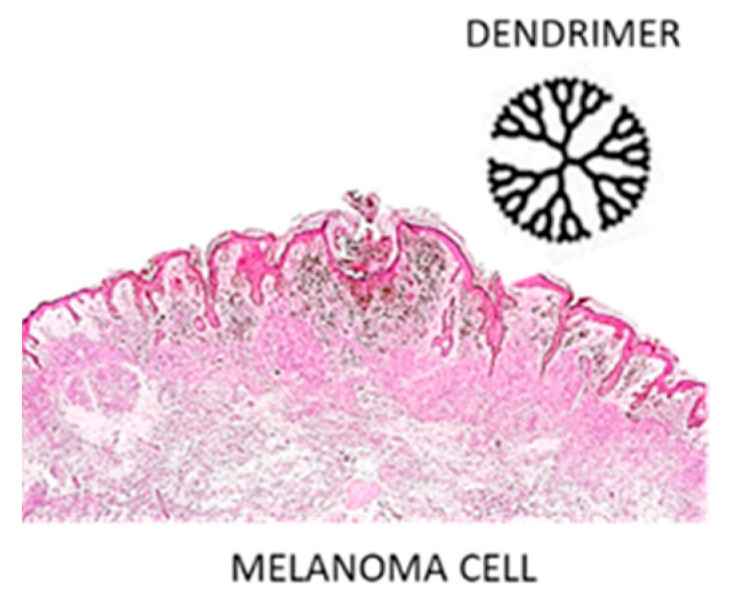
Melanoma cell and dendrimer structure.

Dendrimers are molecules with a central core and repeated branches. They are classified on the basis of its form as polymers, hyperbranched polymers, or brush-polymers and also can be classified by their molecular weight as low or high molecular weight [68]. There are various applications of these molecules in the biomedical and pharmaceutical fields, in particular dendrimer nanoparticles can be functionalized with varied ligands to reach the tumor tissue through different barriers in the body with minimal loss of activity in the bloodstream. Therefore, these molecules have the capacity to selectively kill tumor cells without affecting healthy ones [69]. Dendrimers are particularly useful for melanoma treatment (Figure 5).

In addition, anticancer drugs encapsulated in dendrimers exhibit slower release, lower toxicity, and higher accumulation in solid tumors compared to free drugs [70].

With the aim to generate nanosystems’ able to kill cancer cells, Tassano et al. decided to combine nanoproperties of dendrimers and therapeutic capacity of radionuclide ^188^Re [71]. Their idea has been due to literature data about the use of dendrimers as vehicles of beta radionuclides that described their potential therapeutic use [72]. In addition, active molecular agents bound to ^188^R, have also been used, such as for example the analogous peptides, for the treatment of melanoma through targeted irradiation of tumor while minimizing the dose to the kidneys [73]. Hence Tassano and his collaborators demonstrated that ^188^Re-dendrimer can produce double-stranded breaks in DNA inducing chromosomal aberration in melanoma cells in vitro.

More recently Xia et al., developed a cationic polyamidoamine dendrimer (amino-terminated PAMAM) as promising vehicle for simultaneous delivering of doxorubicin (DOX) and immunoadjuvant cytosine-phosphate-guanine oligonucleotides (CpG ODNs) to treat metastatic melanoma [74]. In particular, DOX was conjugated on the amino-terminated PAMAM dendrimer by pH-sensitive hydrazone bond (PPD). The authors also confirmed that the synthesized PPD conjugates had favorable properties to load nucleic acids. Besides the low molecular weight heparin (LMWH) was also integrate providing not only anti-metastatic effects, but also shielding the positive charge of PPD/CpG, helping to reduce the clearance by the reticuloendothelial system (RES) for better tumor accumulation and reduce the potential toxicity of cationic nanoparticles. Therefore, this combination of chemotherapy and immunotherapy based on this multifunctional core–shell nanoplatform showed enhanced treatment efficiency on melanoma primary tumor and pulmonary metastasis.

### 3.6. Solid Lipid Nanoparticles and Nanostructured Lipid Carriers

**Figure 6:**
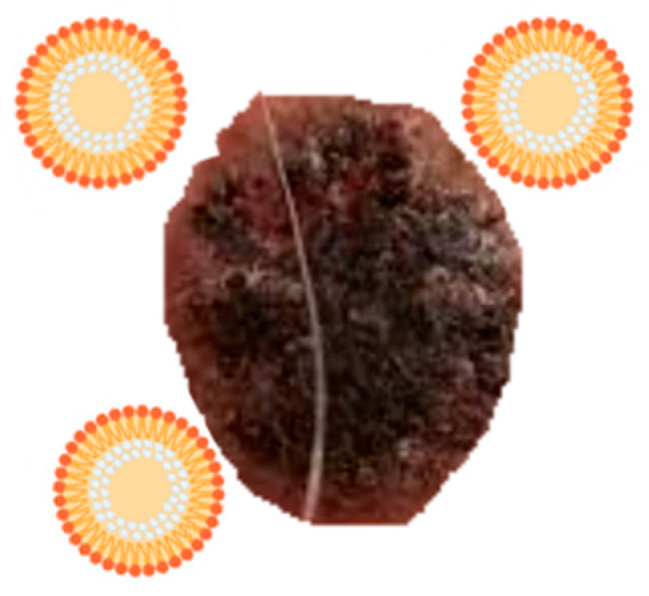
Solid lipid nanoparticles’ (SLNs’) interaction with melanoma.

Solid lipid nanoparticles (SLNs) are colloidal particles of submicron size, with a diameter between 50 and 1000 nm (Figure 6).

They are made of a lipid matrix solid at physiological temperature, surfactants and, sometimes, by cosurfactants. In addition, they less toxic and biocompatible compared to inorganic or polymeric nanoparticles [75,76,77]. Nanostructured lipid carriers (NLCs) are drug-delivery systems composed of both solid and liquid lipids as a core matrix. Both NLCs and SLNs have attracted increasing attention in recent years as drug delivery systems [78].

Clemente et al. developed solid lipid nanoparticles (SLNs) that encapsulated the anti-cancer drug Temozolomide (TMZ), suitable for intravenous administration, useful for the treatment of melanoma [79]. The idea of including TMZ in SLNs was due to the possibility of overcoming its side effects and improving its properties therapeutic properties. Compared to TMZ free, SLNs-TMZ exerted greater effects, when cell proliferation of melanoma cells and neoangiogenesis were both evaluated. In particular, SLNs containing TMZ inhibited growth and vascularization of B16-F10 melanoma in C57/BL6 mice, without evident toxic effects. As result solid lipid nanoparticles could be a promising strategy for TMZ delivery, allowing for an increase stability of the drug and therefore its use in the treatment of aggressive malignant tumors.

Banerjee and coauthors fabricated solid lipid nanoparticles, modified with Tyr-3-octreotide (PSM), to treat melanoma that highly expresses somatostatin receptors (SSTRs) [80]. PSM demonstrated greater anti-melanoma, anti-migratory and anti-invasive activity in the B16F10 cell line compared to dacarbazine (DTIC), a chemotherapy drug used for aggressive melanoma. Furthermore, PSM showed favorable tumor accumulation after local and systemic administration by effectively inhibiting melanoma in mouse models of subcutaneous and experimental lung metastases. In particular, PSM therapy generated an immunogenic environment in the tumor contributing to the production of systemic antitumor immunity that improved the therapeutic efficacy of PSM compared to DTIC. All this may offer a new perspective for the therapy of SSTR2 positive metastatic tumors.

In their work, Malta et al. produced lipid nanoparticles (NLC) encapsulating LEM2, a synthetic xanthone with potent antiproliferative effect towards A375 melanoma cells [81]. The idea to encapsulate LEM2 into the nanoparticles, was due to its poor aqueous solubility, related to poor bioavailability, limiting its therapeutic use. The NLC, produced by either ultra-sonication or hot HPH, showed good and similar particle sizes and appeared stable over 60 days. During the production of unloaded NLCs, it was noted that the ultrasonication method was easier and faster when compared with hot HPH. For this reason, LEM2 was encapsulated in NLCs using the ultrasonication method. Loaded NLCs were more cytotoxic against melanoma A375 cell line than unloaded NLC, possibly due to LEM2 antitumor activity, which means that NLCs could be used as a nanocarrier to this drug, improving its delivery problems.

### 3.7. Microneedles

**Figure 7:**
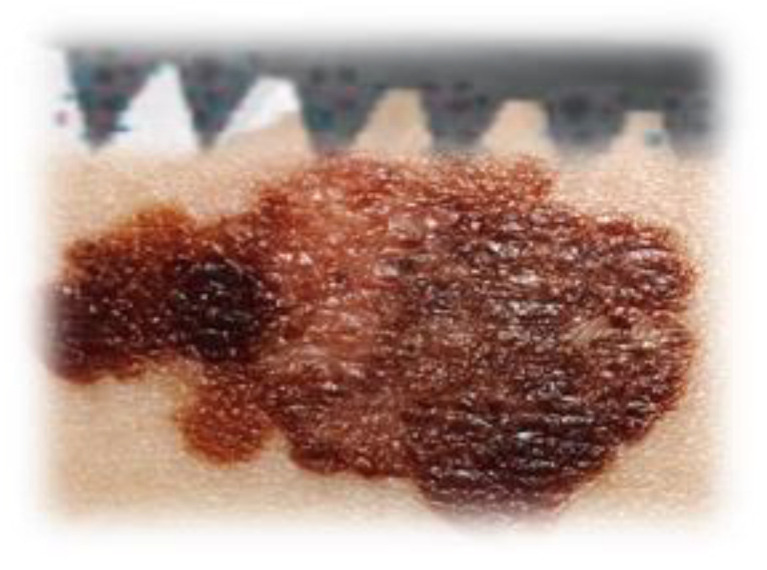
Microneedles toward melanoma.

Microneedles (MNs) technology is an interesting strategy for transdermal drug delivery that has emerged in recent years [82] (Figure 7).

Their size is very small and the length of the needle is usually less than 1 mm, practically invisible to the naked eye [83]. Furthermore, the MNs only penetrate the epidermal layer without damaging the neurons and capillaries in the dermis, thereby minimizing the pain associated with transdermal administration and without causing skin damage and bleeding [84]. Huang et al. has first synthesized a dextran methacrylate hydrogel (DexMA) to develop a new type of NMs for continuous transdermal administration of doxorubicin (DOX) and trametinib (Tra) two FDA-approved anticancer drugs [85]. The antitumor efficacy in nude mice xenograft of B16 cells was evaluated by highlighting the synergistic anticancer effects of DOX and Tra. Furthermore, the authors confirmed that the prepared MNs possessed sufficient mechanical strength and produced good continuous drug release. MNs loaded with DOX and Tra could represent a synergistic treatment of skin cancer.

Quin and colleagues, recently, developed dissolution microneedles (DMNs) packed in solid lipid nanoparticles (SLNs) useful for melanoma therapy [86]. The anticancer drug paclitaxel (PTX) and the photothermal agent IR-780 were co-loaded into heat-sensitive SLNs to achieve multiple time-controlled doses in a single administration. SLNs were placed on the needle tips of the DMNs to achieve accurate delivery. After distribution of PTX/IR-780 SLNs into the tumor site, an irradiation was applied and, consequently, IR-780 absorbed light energy and converted it into heat, leading to an in situ phase transition of SLNs that caused PTX burst release. Once the laser was switched off, the temperature decreased, SLNs were re-solidified, and the PTX release was narrow. In subsequent analyses, the pharmacodynamics of the formulations were evaluated in vitro and in vivo. Thus the obtained PTX/IR-780 SLNs @DMNs could provide multiple chemo-photothermal therapies in a single administration resulting also more effective, in inhibiting tumor growth, respect to intravenous and intratumoral injections of PTX/IR-780 SLNs.

## 4. Nanotechnologies and Immunotherapy in Melanoma

A crucial role of the immune system is its ability not to attack normal body cells. To do this, it uses “checkpoints”, which are proteins found on immune cells that must be activated (or deactivated) to induce an immune response. Melanoma cells also sometimes use these checkpoints to avoid being attacked by the immune system. But several drugs block precisely the checkpoint proteins, helping to restore the immune response against melanoma cells. In fact, so-called checkpoint inhibitors showed an improved response rates, a better long-term duration of control, and a longer overall patient survival [87,88].

In this context, nanoparticles and biomaterials are particularly useful since are enable to program the localization, pharmacokinetics, and co-delivery of immunomodulatory compounds, eliciting responses that cannot be achieved upon administration of such compounds in solution [89]. Nanoparticles may help to improve efficacy of various immunotherapies and increase the number of patients who achieve long-term remission [90].

Cai and co-authors, in their recent review described engineered biomaterials as scaffolds in implantable, injectable, or transdermal delivery devices to allow tunable drug release kinetics and delivery for up to weeks at lower drug doses. These biomaterial scaffolds can be loaded with chemical agents, cells, tumor-associated antigens, and/or adjuvants that directly activate the immune system or that modular the tumor microenvironment [91].

Also the induction of adequate immune responses after photo tumor ablation may be fundamental to obtain a long term therapeutic effect of phototherapy. For this reason, Zhu et al. projected the photosensitizer chlorin e6 (Ce6) and the well-known immunoadjuvant aluminum hydroxide into bovine serum albumin by biomineralization as a novel nanosystem (Al-BSA-Ce6 NPs) [92]. After intravenous injection, the nanoparticles were able to destroy tumor cells effectively and, at the same time protect animals against tumor rechallenge and metastasis by strongly inducing a systemic anti-tumor immune response. In addition, T cells accumulated in lymph nodes and infiltrated the tumor site, elevating levels serum antibody, cytokine level, cytotoxic T cells, and Th1 cells. These protective effects were not observed with commercially available alumina gels, or when the aluminum hydroxide in the nanoparticles was replaced with ferric hydroxide. Therefore, obtained result showed Al-BSA-Ce6 NPs as a novel and unique system for alumina adjuvants useful instrument for cancer therapy.

Zhang et al. in a letter to editor, highlighted the use copper-cysteamine (Cu–Cy) nanoparticles in the treatment of melanoma [93]. In particular, they demonstrated that nanoparticles mediated photodynamic therapy (PTD), could induce potent antitumor immune responses via dendritic cells maturation; subsequent activation of CD4+T cells, CD8+T cells, and NK cells which inhibited tumor growth by killing or suppressing tumor cells. In addition, due to X-ray activation, Cu–Cy nanoparticles can effectively induce an antitumor immune response caused to the produce substantial levels of ROS that lead to the direct destruction of melanoma.

**Figure 8:**
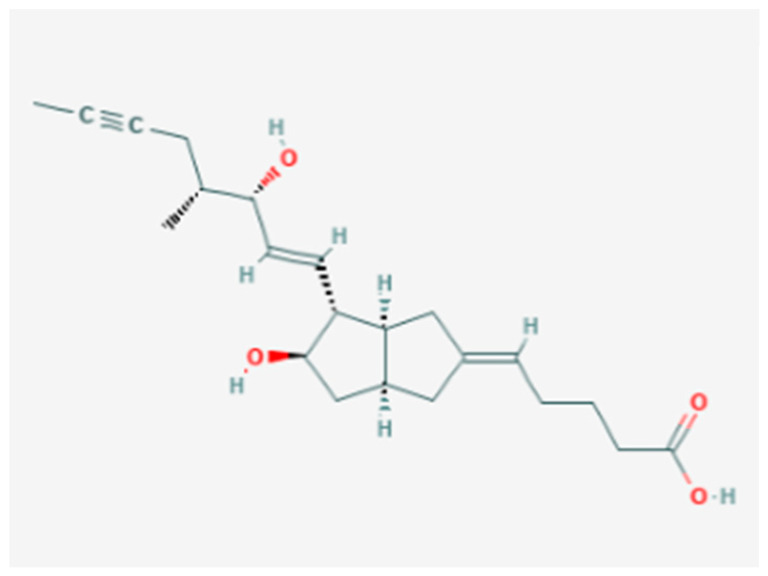
Interleukin-2 chemical structure.

An important role in immunotherapy is played by Interleukin-2 (IL-2), it is a cytokine mainly produced by activated B or T lymphocytes (Figure 8).

IL 2 has been available for decades in the treatment of melanoma but its systemic use is limited by severe systemic side effects and disappointing complete responses of only 10% of patients [94]. There are several examples of chemical modifications of IL-2 or construction of innovative materials for its delivery in the therapy of advanced melanoma.

In order to reduce the severity of IL-2-associated adverse effects, Xie et al. proposed an interesting approach [95]. In particular, they developed a delivery system by binding redox-sensitive IL-2/Fc nanogels to the surface of adoptively transferred T cells. Hydrogel systems have received increasing attention in recent years as materials for drug delivery systems (DDS), because they are biocompatible and non-toxic [96]. They consist of three-dimensional, hydrophilic polymeric networks able to absorb large quantities of water or biological fluids, due to the presence of hydrophilic groups, and to release the drugs trapped in them through a slow diffusion. They possess modular physical properties and the ability to protect labile drugs from degradation by controlling their release [73]. Interestingly, cytokine-containing nanogels are capable of selectively release IL-2 in response to activation of T cell receptors. It was demonstrated in a mouse model of melanoma that this delivery system had better efficiency than free IL-2, without evident toxicity [97].

Zhang et al. [98] combined IL-2 with the antibody against the T cell receptor CD137 inside immunoliposome delivery systems. They observed these immunoliposomes showed an equivalent antitumor activity as compared to the free components, and low or no systemic toxicity. This beneficial effect was related to the attachment of IL-2 and anti-CD137 to the surface of liposomes that allowed a rapid accumulation of immunoliposomes in melanoma, thus, minimizing the systemic utilization, as well as the risk of toxicity.

Interleukin-12 (Il-12), a heterodimeric cytokine, has also been shown to possess anticancer effects in various animal models. However, the main problem associated with the release of this protein is its instability and cytotoxicity following systemic administration in rodents and in clinical studies. Therefore, gene delivery systems based on nanoparticles could be effective in this sense, both for the high penetrability of the tissues and for their cellular uptake [99].

### Nanovaccines

Nanovaccines may represent an interesting strategy in the field of cancer immunotherapy. In this context Xu et al. described a procedure to fabricate a custom nanovaccine, based on a cationic fluoropolymer, useful for post-surgical immunotherapy of cancer [100]. Nanoparticles formed by mixing the fluoropolymer with an ovalbumin template antigen, dendritic cell maturation via the Toll-like receptor 4 (TLR4)-mediated signaling pathway, and promoted antigen transportation into the cytosol of dendritic cells, which leads to an effective antigen cross-presentation. This nanovaccine was able to inhibit ovalbumin-expressing B16-OVA melanoma.

Zeng and collaborators developed a glycosylated gold nanoparticles/peptides nanovaccine based on non-covalent interaction between β-cyclodextrin (β-CD) modified gold nanoparticles and Adamantanecarboxylic acid (AD) linked with antigens [101]. This nanovaccine can generate significant titers of antibodies with aN improved immune response as well as therapeutic effect against melanoma, suggesting, particularly, that the immunogenicity of peptide antigens could be improved by loading with this carrier.

Another interesting approach was used by Conniot et al. who demonstrated that NPs could be used in the treatment of melanoma, similar to the use of vaccines for viral diseases [102]. In particular, the NPs, made of biodegradable polymer, were individually packaged with two peptides usually expressed in melanoma cells: Melan-A/MART-1 (26–35 (A27L) major histocompatibility complex class I (MHCI) restricted peptide (MHCI-ag), and the limited peptide MHCII Melan-A/MART-1 (51–73) (MHCII-ag), directed towards the MHC class I and class II antigen presentation pathways. Furthermore, the NPs were also grafted with the mannose receptors, making them capable of being mediated by the ligand (active target of dendritic cells). Mannosylated NPs were combined with an anti-PD-1 (αPD-1) antibody for blocking immunosuppression or with an anti-OX40 antibody (αOX40) for T cell stimulation. After injection of the nanovaccines into mouse models, they showed both a prophylactic effect, preventing the development of melanoma in healthy immunized mice, and a therapeutic effect, delaying the progression of the disease in mice of advanced melanoma.

## 5. Molecular Targeted Therapy

About half of all melanomas carry a specific mutation in the BRAF and MEK genes known as V600E and V600K, respectively [103]. In this regard, in recent years the combination of BRAF and MEK inhibitors has been the treatment of choice for people with advanced melanoma which allows them to live longer, without disease progression, when treated with combinations of BRAF and MEK compared to treatment with chemotherapy or a single inhibitor of BRAF or MEK. On the other hand, the therapy based on BRAF/MEK inhibitors (MAPKi) is conditioned by drug resistance. In this context, Fattore et al. recently discovered that several microRNAs are involved in the development of this drug resistance [104]. In particular, it seems that miR-204-5p and miR-199b-5p function as resistance antagonists because their overexpression is able to inhibit the growth of melanoma cells in vitro both alone and in combination with MAPKi. However, miRNAs are readily degraded in serum and biological fluids, and are also characterized by poor intracellular absorption. Therefore, Fattore and collaborators developed lipid nanoparticles (LNPs) that encapsulate miR-204-5p, miR-199b-5p alone or in combination. These formulations have been tested in vitro on several MAPKi-sensitive melanoma cell lines or made drug resistant. The obtained results showed that the LNPs that encapsulate the combinations of the two tumor suppressor miRNAs are highly efficient in inhibiting the proliferation and viability of melanoma cells, and potentiate the efficacy of drugs that inhibit BRAF and MEK.

Also of interest was the work of Ruan et al., who, for the first time, developed a BRAF (siBraf) siRNA delivery system based on cell-penetrating octaarginine (R8) peptide nanocomplexes combined with coated microneedles (MN). [105] In particular, the NMs were coated with R8/siBraf, in order to obtain a targeted anti-melanoma treatment. In vitro experiments on cell lines have shown that R8/siBraf can enhance siBraf transfection, silence the BRAF gene and inhibit tumor cell growth. NMs coated with R8/siBraf can also effectively release R8/siBrafin at the pathological site. In vivo experiments indicated that R8/siBraf-coated MNs can significantly inhibit the development of melanoma, induce apoptosis of tumor cells and suppress their proliferation. The BRAF gene in the tumor was also significantly silenced in vivo. The intradermal release of SiBraf via the combination of NM and R8 nanocomplexes could be also a promising approach for the treatment of cutaneous melanoma, which exploits both the characteristics of MN and the cell penetrating peptide to achieve the efficacy of targeted inhibition on cutaneous melanoma.

## 6. Chemotherapy

Chemotherapy is the treatment used for most cancers due to its undisputed efficacy [102]. Unfortunately, the poor solubility and permeability of most chemotherapy drugs results in very low bioavailability [106]. In this context, nanotechnology appears to be advantageous for encapsulating lipophilic drugs, improving drug penetration depth, and achieving a specific target effect [107].

With the aim to enhance tumor penetration capability of 10-Hydroxycamptothecin (HCPT) and improve the chemotherapeutic effect of melanoma, Guo et al. developed positively-charged nanoparticles based on chitosan (NPs/HCPT) [108]. HCPT was encapsulated into the core of nanoparticles and this strategy significantly improved the aqueous dispersibility and permeability. The sustained release of HCPT from NPs/HCPT in the cytoplasm maintained an effective drug concentration, thus effectively inhibiting the proliferation of cancer cells.

The obtained results suggested that cationic NPs/HCPT could be potentially applied as promising chemotherapies delivery nanosystem.

In a recent article Adhizabe et al., described the efficiency of titanium dioxide (TiO_2_) nanoparticles (NPs) to enhance the effects of chemotherapy in vitro and in vivo models of murine melanoma [109]. Therefore, F10 melanoma cells were exposed to various concentrations of TiO_2_ NPs and/or cisplatin, then cell growth, cell viability, and cell death were evaluated. In addition, C57BL/6 melanoma mice were treated by TiO_2_ NPs and/or cisplatin, and then drug responses, tumor size, and mouse organs were studied. The results showed that nontoxic concentrations of TiO_2_ NPs, equal to, 50 µg/mL, can promote anti-proliferative and cytotoxic effects of cisplatin in F10 melanoma cells. Furthermore, the TiO_2_ combination with cisplatin produced a higher inhibition of tumor growth compared with each monotherapy.

Dianzani and collaborators proposed a new nanotechnology-based poly-chemotherapy [110]. In particular, they employed temozolomide (TMZ), rapamycin (RAP), and bevacizumab (BVZ) co-loaded in injectable nanoemulsions, for parenteral nutrition (Intralipid^®^), useful in the treatment of advanced stage melanoma. These drugs, efficiently loaded in the liquid lipid matrix of Intralipid^®^, leaded to a fast internalization in tumor cells, an increased cytotoxicity towards melanoma cells, as well as an improved inhibition of tumor relapse, migration, and angiogenesis as demonstrated in cell models. In vivo studies showed that the proposed poly-chemotherapy was able to reduce tumor growth significantly, compared to controls. The obtained results indicated that Intralipid^®^ could represent a safe and versatile delivery system for advanced melanoma treatment.

## 7. Conclusions

Melanoma is a highly aggressive malignancy that is very hard to treat due to its multi-drug resistance. In addition, conventional chemotherapy and immunotherapy are limited by low response rates and show no improvement on overall survival. In the last years, among various adopted strategies, there are the nanoparticles (NPs which hold great promise to improve antitumoral drugs’ delivery because they can increase efficacy while at the same time reducing the side effects of conventional formulations. This review aimed to describe the recent and innovative studies regarding NPs for drug delivery in melanoma treatment, highlighting their important role in the improvement of prognosis of this pathology for the future.

## Data Availability

Not applicable.
